# Periodontitis related to cardiovascular events and mortality: a long-time longitudinal study

**DOI:** 10.1007/s00784-020-03739-x

**Published:** 2021-01-28

**Authors:** Viveca Wallin Bengtsson, Gösta Rutger Persson, Johan Sanmartin Berglund, Stefan Renvert

**Affiliations:** 1 University of Kristianstad, Elmetorpsvägen 15, 29188 Kristianstad, Sweden; 2 Department of Periodontics, University of Washington, Seattle, WA USA; 3 Departments of Periodontics and Oral Medicine, University of Washington, Seattle, WA USA; 4 Department of Health, Blekinge Institute of Technology, Karlskrona, Sweden; 5 Department of Clinical Sciences, Lund University, Lund, Sweden; 6 Dublin Dental Hospital Trinity College, Dublin, Ireland; 7 Faculty of Dentistry, The University of Hong Kong, Hong Kong SAR, China

**Keywords:** Periodontitis, Ischemic heart disease, Mortality, Epidemiology

## Abstract

**Objective:**

The present study assessed if individuals ≥ 60 years of age with periodontitis are more likely to develop stroke or ischemic heart diseases, or at a higher risk of death for 17 years.

**Material and methods:**

At baseline individuals ≥ 60 received a dental examination including a panoramic radiograph. Periodontitis was defined as having ≥ 30% sites with ≥ 5-mm distance from the cementoenamel junction to the marginal bone level. Medical records were annually reviewed from 2001 to 2018. Findings from the medical records identifying an ICD-10 code of stroke and ischemic heart diseases or death were registered.

**Results:**

Associations between periodontitis and incidence of ischemic heart disease were found in this 17-year follow-up study in all individuals 60–93 years (HR: 1.5, CI: 1.1–2.1, *p* = 0.017), in women (HR: 2.1, CI: 1.3–3.4, *p* = 0.002), and in individuals 78–96 years (HR: 1.7, CI: 1.0–2.6, *p* = 0.033). Periodontitis was associated with mortality in all individuals (HR: 1.4, CI: 1.2–1.8, *p* = 0.002), specifically in men (HR: 1.5, CI: 1.1–1.9, *p* = 0.006) or in ages 60–72 years (HR: 2.2, CI: 1.5–3.2, *p* = 0.000). Periodontitis was more prevalent among men (OR: 1.8, CI: 1.3–2.4, *p* = 0.000).

**Conclusions:**

Individuals with periodontitis have an increased risk for future events of ischemic heart diseases and death.

**Clinical relevance:**

Improving periodontal health in older individuals may reduce overall mortality and ischemic heart diseases. Both dental and medical professionals should be aware of the associations and ultimately cooperate.

## Introduction

Periodontitis is a chronic disease with an infectious etiology, causing an inflammatory response resulting in the breakdown of soft and hard tissues around teeth [1]. Severe periodontitis has been identified as the sixth most prevalent disease in the world [2]. In the USA, the prevalence of periodontitis is reported to be 47% [3]. In older individuals, it is even more prevalent [4].

Cardiovascular diseases (CVDs) include all diseases associated with the heart and blood vessels, such as stroke, coronary heart disease, and heart failure [5]. CVDs are the most common causes of death in the USA [6]. Atherosclerosis is considered the leading cause of CVDs [7, 8]. Periodontal infections may cause bacteremia triggering host systemic inflammatory responses and chronic inflammation and related to the pathogenesis of atherosclerosis [9]. Data suggest that periodontitis is associated with subclinical atherosclerosis [7, 8, 10].

Periodontitis has been associated with an increased risk for CVDs [8, 11–13]. Having periodontitis has been reported to increase the risk for stroke [14–16]. Periodontal disease has been pointed out as a risk factor for stroke, especially in men and in younger subjects [17]. In a review and meta-analysis of the literature, periodontitis was reported to be associated with the occurrence of stroke [18]. Periodontitis has also been associated with myocardial heart infarction [19, 20]. Individuals diagnosed with the first event of acute myocardial infarction (AMI) were matched with subjects with no evidence of AMI. A clear association was reported between periodontitis and AMI [19]. In another control-matched study by Rydén et al. 2016[20], an increased risk for a first myocardial infarction was reported among individuals with periodontitis. In a review by Dietrich et al. [21], six case-control and cohort epidemiological studies described an increased risk for a first coronary event in individuals with diagnosed periodontitis. In general, epidemiologic data are linking periodontitis to CVDs. Periodontitis is presently believed as a CVD risk factor. Several studies assessing associations between periodontitis and CVDs are however cross-sectional cohort or case-control studies [7, 8, 16]. Few studies with a prospective longitudinal study design have been reported.

Accordingly, the present study aimed to assess if individuals ≥ 60 years of age with periodontitis are more likely to develop stroke or ischemic heart diseases or are at a higher risk of death over 17 years.

## Material and methods

### Study individuals

Inclusion criteria selected the study individuals from the Swedish National Study of Aging and Care (SNAC). SNAC is a population-based, prospective longitudinal study in which SNAC-Blekinge is one participating research center. At the baseline in 2001–2003, an equal number of study individuals in age cohorts of 60, 66, 72, and 78 were randomly selected from the Swedish population database for Karlskrona City (The Swedish Tax Agency electronic database) and were invited by regular mail. All individuals in the community at age 81, 84, 87, 90, 93, and 96 years were also invited to participate at baseline, representing the older population in Karlskrona, Sweden. In total, 1402 individuals agreed to participate. All participants signed an informed consent. The principles of the Helsinki declarations were followed. The Ethics Committee Lund, Sweden approved the study (LU 604-00, LU 744-00). Baseline inclusion criteria were as follows: (i) age between 60 and 96 years and living in the community of Karlskrona, Sweden, (ii) dentate, with one or more teeth. Exclusion criterion was (i) non-readable panoramic radiographs.

Medical and dental research teams at a research center in Karlskrona, Sweden, examined the study participants. The overall response rate was 62%, representing approximately 10% of the entire population ≥ 60 years of age in the community. The proportion per group was 53% in individuals 60–78 years (the randomly selected), and the response rate in 81–96 years was 47%.

### Radiographic measurement

An analogue panoramic radiograph using a standard exposure of 75 kV/10 mA was obtained using an Orthopantomograph (OP 100, Instrumentarium, Tuusula, Finland; film Kodak T-Mat G/RA, intensifying screen Kodak Lanex Regular, film processor Durr XR 24). The radiographic measurements were made from the panoramic radiographs exposed at baseline between 2001 and 2003. Among the initial 1402 radiographs, 858 readable radiographs meeting the inclusion criteria of presenting with at least one tooth were included in the present study.

An independent, experienced examiner (REP) performed the radiographic measurements. The examiner was masked to medical conditions, gender, age, and survival status of the study individuals. Bone loss was measured, based on the number of interproximal sites, as percent loss of bone from the enamel cement junction (CEJ) to the highest marginal bone level on the mesial and distal surfaces of each tooth. Alveolar bone loss ≥ 5-mm distance from CEJ to marginal bone level on ≥ 30% of sites was used as the definition of periodontitis. Intra-class coefficient (ICC) analysis between randomly selected cases for double assessments regarding the reproducibility of the distance between CEJ to the apex was 0.93 (95% CI: 0.91–0.96, *p* < 0.01) between the first and second reading.

### Medical examination

Cerebrovascular diseases (stroke) and ischemic heart diseases were registered from an electronic medical database at the research center of the general hospital in Karlskrona and following the International Statistical Classification of Diseases and Related Health Problems 10th revision ICD-10 codes (ICD-10): ICD *I60-69* for stroke and ICD *I20-25* for ischemic heart diseases. A physician (JB) annually reviewed the medical database, including all medical records between 2001 and 2018, assessing medical records of the participating individuals in the 17 years following the baseline examination. Any findings from the medical records identifying an ICD-10 code were identified as a positive finding. Death or the first event of a stroke or ischemic heart disease was recorded as an event. Information on diabetes type 2, hypertension, or smoking was identified from a self-reported questionnaire on medical history at baseline, with a focus on a history of acute myocardial infarction (AMI) or a history of stroke. Smoking included both current and former smoking versus non-smoking included only those individuals with no current nor former history of smoking.

### Statistics

The Statistical Package for the Social Sciences (SPSS) Predictive Analytics Software (PASW) 25.0 statistical software package (SPSS Inc., Armonk, NY, USA) for personal computer (PC) was used in the analyses. The data were analyzed using descriptive and inferential statistics. Dichotomous data were analyzed using the Pearson *χ*^2^ test, and by Mantel–Haenszel common odds ratio. Survival statistics with Cox regression analysis, method enter, was used to study adjusted associations. A multivariable adjustment was made for age, body mass index (BMI) ≥ 30, diagnosis of diabetes mellitus type 2, gender, hypertension, history of acute myocardial infarction (AMI), history of stroke, periodontitis, and smoking. Proportional hazards assumption was evaluated graphically with “log-log” plots. Time was defined as months from inclusion (dental examination) to either stroke, respectively ischemic heart diseases or death outcome or censoring due to emigration, death, or end of follow-up. Statistical significance was declared at *p* < 0.05.

## Results

### Demographic data

Data were derived from 858 individuals (women 53.5%). During the 17-year follow-up period, 492/858 (57.3%) died, and 51/858 (5.9%) moved away. The ages at baseline varied between 60 and 93 years with median age 72.0 years. On average, the individuals had 18.6 remaining teeth (SD: ± 7.5). Approximately half of the individuals 428/838 (51.1%) reported that they did not or had never smoked. At the baseline examination, periodontitis was declared in 212/858 (24.7%) (Table 1). Men had a higher prevalence of periodontitis than women, 121/212 (57.1%) (OR: 1.8, CI: 1.3–2.4, *p* = 0.000). Data were derived from 858 individuals (women 53.5%) with 471/858 (54.9%) in ages 60-72 years, young old (YO), and 378/858 (45.1%) in ages 78-96 years, old-old (OO) 78–96 years age group, including 471/858 (54.9%) respectively 387/858 (45.1%) individuals.

**Table 1:** Baseline 2001–2003, characteristics of the study individuals

	≥ 1 remaining tooth, dental examination, readable panoramic radiograph *n* = 858
Variables	
Median age (years)	72.0 (SD: ± 9.3)
Gender (women)	53.5%
Mean remaining teeth	18.6 (SD: ± 7.5).
Periodontitis	212/858 (24.7%)
No current nor former smoker	428/838 (51.1%)
BMI ≥ 30	192/853 (22.4%)
Hypertension	260/847 (30.3%)
History of stroke	35/853 (4.1%)
History of AMI	66/850 (7.7%)
Diabetes type 2	66/856 (7.7%)

*AMI* acute myocardial infarction, *BMI* body mass index, Periodontitis = alveolar bone loss ≥ 5-mm distance from the CEJ to marginal bone level on ≥ 30% of sites. Self-reported at baseline: no current nor former smoker, hypertension, history of stroke, history of AMI, and diabetes type 2

### Periodontitis and incidence of ischemic heart diseases or stroke during the follow-up period

The cumulative incidence of ischemic heart diseases between 2001 and 2018 was 203/858 (23.7%), with men 102/203 (50.2%) and women 101/203 (49.8%) (OR: 1.2, CI: 0.9–1.7, *p* = 0.221). The incidence of ischemic heart diseases was 57.2 incidences per year and 6668 per 100,000 and year. The cumulative incidence of stroke was 118/858 (13.8%), with 60/118 (50.8%) men respectively 58/118 (49.2%) women (OR:1.2, CI: 0.8-1.8, p=0.308). Stroke incidence was 24.86 per year, which corresponds to 2898 strokes per 100,000 persons and year.

**Fig. 1:**
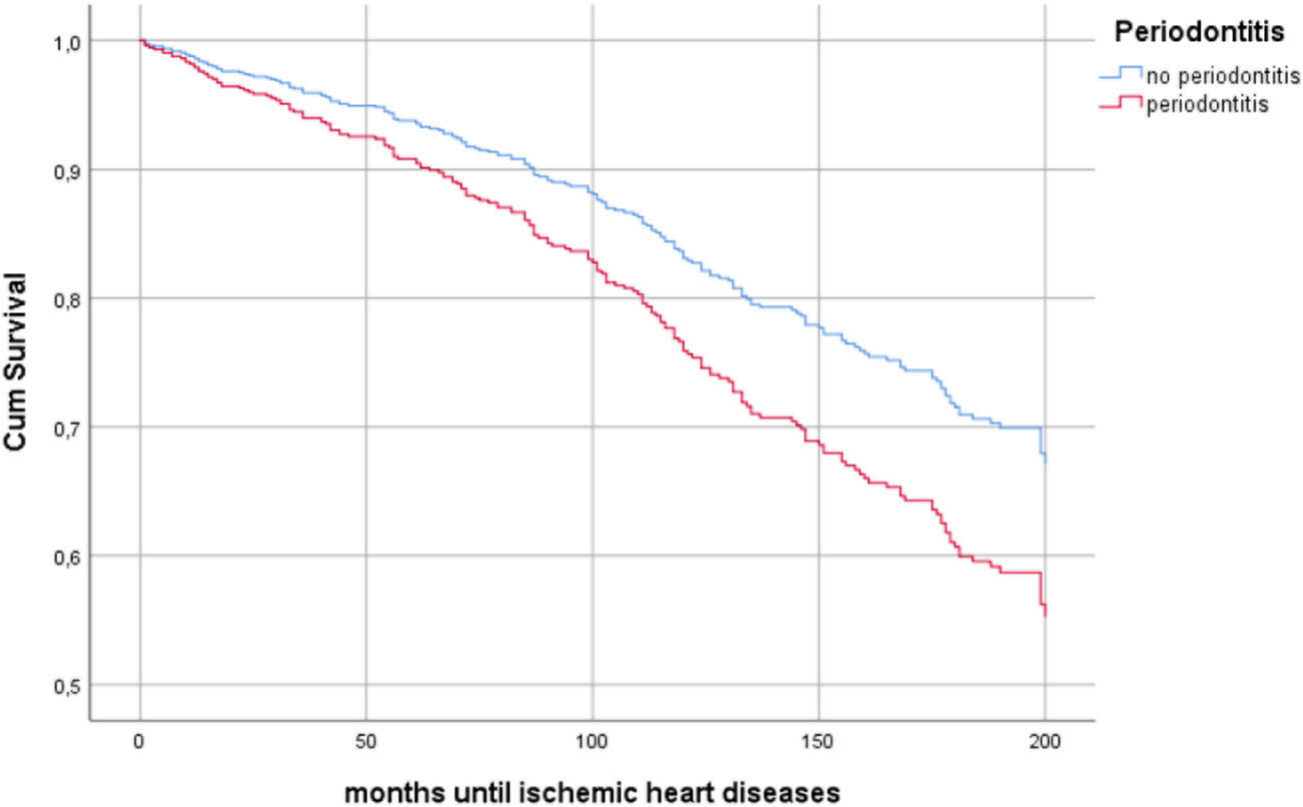
Cox regression curves: 17-year cumulative ischemic heart disease survival of the total population, comparing individuals with and without periodontitis

Cox regression analysis based on baseline data with periodontitis as an independent variable and incidence of the first event of a stroke or ischemic heart diseases as the dependent variable and with adjustment for the variables age group, BMI ≥ 30, diabetes type 2, gender, hypertension, history of acute myocardial infarction, history of stroke, and smoking, was used. Periodontitis increased the risk for ischemic heart diseases in all individuals (HR: 1.5, CI: 1.1–2.1, *p* = 0.017) (Fig. 1), in women (HR: 2.1, CI: 1.3–3.4, *p* = 0.002), and in the OO group (HR: 1.7, CI: 1.0–2.6, *p* = 0.033) (Table 2). No significant association was identified between periodontitis and stroke, in neither of all individuals, women, men, YO, and OO (Table 3).

**Table 2:** Associations between ischemic heart diseases and different independent variables including periodontitis by Cox regression analysis

Independent variables at baseline	All individuals (*n* = 858) HR, CI, *p* value	Women (*n* = 459) HR, CI, *p* value	Men (*n* = 399) HR, CI, *p* value	YO (*n* = 471) HR, CI, *p* value	OO (*n* = 387) HR, CI, *p* value
Age category	HR: 1.9, CI: 1.4–2.5, *p* = 0.000**	HR: 2.5, CI: 1.6–3.8, *p* = 0.000**	HR: 1.4, CI: 0.9–2.2, *p* = 0.098		
BMI ≥ 30	HR: 1.2, CI: 0.9–1.7, *p* = 0.246	HR: 1.2, CI: 0.8–2.0, *p* = 0.417	HR: 1.3, CI: 0.8–2.2, *p* = 0.304	HR: 1.3, CI: 0.8–2.0, *p* = 0.284	HR: 1.2, CI: 0.7–2.1, *p* = 0.479
Diabetes type 2	HR: 1.5, CI: 1.0–2.4, *p* = 0.079	HR: 1.5, CI: 0.8–3.0, *p* = 0.243	HR: 1.4, CI: 0.7–2.6, *p* = 0.310	HR: 1.6, CI: 0.8–2.9, *p* = 0.153	HR: 1.4, CI: 0.7–3.0, *p* = 0.311
Gender	HR: 1.2, CI: 0.9–1.6, *p* = 0.349			HR: 1.4, CI: 1.0–2.2, *p* = 0.079	HR: 0.9, CI: 0.6–1.5, *p* = 0.768
Hypertension	HR: 1.5, CI: 1.1–2.0, *p* = 0.009**	HR: 1.8, CI: 1.2–2.8, *p* = 0.006**	HR: 1.2, CI: 0.7–1.8, *p* = 0.498	HR: 1.5, CI: 1.0–2.3, *p* = 0.049*	HR: 1.5, CI: 1.0–2.3, *p* = 0.079
History of AMI	HR: 2.3, CI: 1.5–3.5, *p* = 0.000**	HR: 2.5, CI: 1.1–5.8, *p* = 0.033*	HR: 2.4, CI: 1.4–4.1, *p* = 0.001**	HR: 2.5, CI: 1.2–5.0, *p* = 0.010*	HR: 2.2, CI: 1.2–4.0, *p* = 0.006**
Periodontitis	HR: 1.5, CI: 1.1–2.1, *p* = 0.017*	HR: 2.1, CI: 1.3–3.4, *p* = 0.002**	HR: 1.1, CI: 0.7–1.7, *p* = 0.749	HR: 1.3, CI: 0.8–2.2, *p* = 0.229	HR: 1.7, CI: 1.0–2.6, *p* = 0.033*
Smoking	HR: 1.1, CI: 0.8–1.5, *p* = 0.634	HR: 1.0, CI: 0.7–1.6, *p* = 0.900	HR: 1.2, CI: 0.8–1.8, *p* = 0.475	HR: 1.3, CI: 0.9–2.0, *p* = 0.181	HR: 0.9, CI: 0.5–1.4, *p* = 0.579

*AMI* acute myocardial infarction, *BMI* body mass index, Periodontitis = alveolar bone loss ≥ 5-mm distance from the CEJ to marginal bone level on ≥ 30% of sites, *YO* young-old 60–72 years, *OO* old-old 78–93 years, *p < 0.0.5, **p < 0.01

**Table 3:** Associations between stroke and different independent variables including periodontitis by Cox regression analysis

Independent variables at baseline	All individuals (*n* = 858) HR, CI, *p* value	Women (*n* = 459) HR, CI, *p* value	Men (*n* = 399) HR, CI, *p* value	YO (*n* = 471) HR, CI, *p* value	OO (*n* = 387) HR, CI, *p* value
Age cut	HR: 3.4, CI: 2.3–5.1, *p* = 0.000**	HR: 3.6, CI: 2.0–6.4, *p* = 0.000**	HR: 3.2, CI: 1.8–5.7, *p* = 0.000**		
BMI ≥ 30	HR: 0.7, CI: 0.4–1.1, *p* = 0.157	HR: 0.7, CI: 0.3–1.4, *p* = 0.282	HR: 0.8, CI: 0.4–1.6, *p* = 0.479	HR: 1.0, CI: 0.5–2.0, *p* = 0.953	HR: 0.5, CI: 0.3–1.1, *p* = 0.098
Diabetes type 2	HR: 0.8, CI: 0.4–1.7, *p* = 0.613	HR: 0.6, CI: 0.2–2.2, *p* = 0.474	HR: 1.0, CI: 0.4–2.7, *p* = 0.924	HR: 0.4, CI: 0.1–1.8, *p* = 0.245	HR: 1.2, CI: 0.5–2.8, *p* = 0.704
Gender	HR: 1.3, CI: 0.9–1.9, *p* = 0.188			HR: 1.2, CI: 0.6–2.2, *p* = 0.587	HR: 1.3, CI: 0.8–2.2, *p* = 0.294
Hypertension	HR: 1.8, CI: 1.2–2.6, *p* = 0.006**	HR: 2.1, CI: 1.2–3.6, *p* = 0.013*	HR: 1.6, CI: 0.9–2.8, *p* = 0.118	HR: 1.7, CI: 0.9–3.1, *p* = 0.128	HR: 1.8, CI: 1.1–3.1, *p* = 0.019*
History of stroke	HR: 3.7, CI: 2.0–6.9, *p* = 0.000**	HR: 2.7, CI: 1.0–7.5, *p* = 0.052	HR: 4.6, CI: 2.1–10.2, *p* = 0.000**	HR: 6.2, CI: 2.7–14.4, *p* = 0.000**	HR: 2.3, CI: 0.9–6.1, *p* = 0.088
Periodontitis	HR: 1.2, CI: 0.8–1.8, *p* = 0.442	HR: 1.3, CI: 0.7–2.4, *p* = 0.485	HR: 1.2, CI: 0.6–2.1, *p* = 0.627	HR: 1.1, CI: 0.5–2.3, *p* = 0.865	HR: 1.2, CI: 0.7–2.0, *p* = 0.535
Smoking	HR: 1.0, CI: 0.7–1.5, *p* = 0.915	HR: 0.8, CI: 0.4–1.4, *p* = 0.381	HR: 1.2, CI: 0.7–2.1, *p* = 0.544	HR: 1.2, CI: 0.7–2.3, *p* = 0.496	HR: 0.9, CI: 0.6–1.6, *p* = 0.834

*BMI* body mass index, Periodontitis = alveolar bone loss ≥ 5-mm distance from the CEJ to marginal bone level on ≥ 30% of sites, *YO* young-old 60–72 years, *OO* old-old 78–93 years, *p < 0.05, **p < 0.01

### Associations to mortality during the follow-up period

**Fig. 2:**
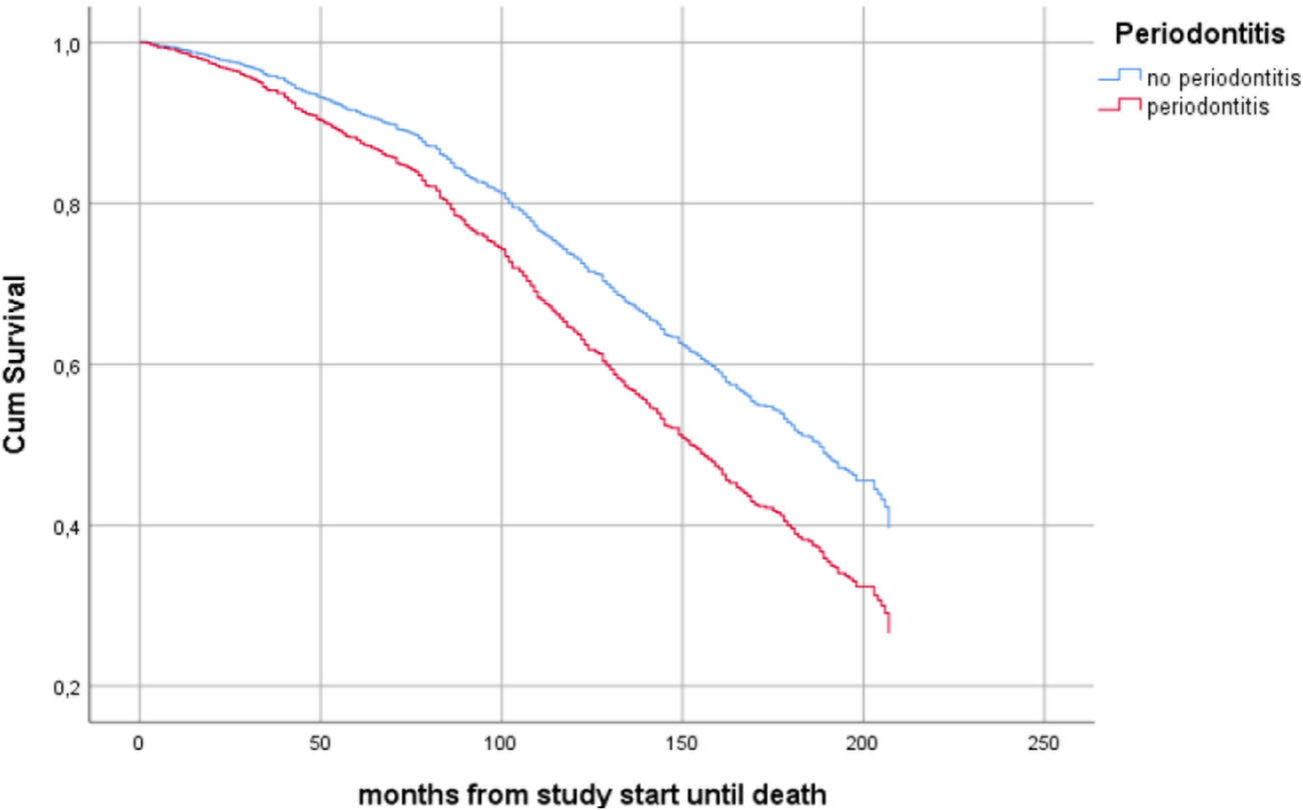
Cox regression curves: 17-year cumulative death survival of the total study population, comparing individuals with and without periodontitis

Data on the cause of death was not available, but during the 17-year follow-up period, 492/858 (57.3%) had died. Among those who had died, 160/492 (32.5%) had periodontitis whereas among those who were alive, 52/366 (14.2%) had periodontitis (OR: 2.9, CI: 2.1–41.1, *p* = 0.000). In individuals with periodontitis, 160/212 (75.5%) (62 women and 98 men) had died and 52/212 (24.5%) (29 women and 23 men) were still alive at the end of the study in 2018 (OR: 1.7, CI: 1.1–2.7, *p* = 0.028). Cox regression analysis with periodontitis as the independent variable and mortality as the dependent variable and with adjustment for the variables age group, BMI ≥ 30, diabetes type 2, gender, hypertension, history of acute myocardial infarction (AMI), history of stroke, and smoking, was used. Periodontitis increased the risk for all-cause mortality in all individuals (HR: 1.4, CI:1.2–1.8, *p* = 0.002) (Fig. 2), in men (HR: 1.5, CI: 1.1–1.9, *p* = 0.006), and in the YO group (HR: 2.2, CI: 1.5–3.2, *p* = 0.000). No associations in women or the OO individuals between periodontitis and mortality were identified (HR: 1.4, CI: 1.0–1.9, *p* = 0.055), respectively (HR: 1.2, CI: 1.0–1.6, *p* = 0.102) (Table 4).

**Table 4:** Associations between mortality and different independent variables including periodontitis by Cox regression analysis

Independent variables at baseline	All individuals (*n* = 858) HR, CI, *p* value	Women (*n* = 459) HR, CI, *p* value	Men (*n* = 399) HR, CI, *p* value	YO (*n* = 471) HR, CI, *p* value	OO (*n* = 387) HR, CI, *p* value
Age cut	HR: 5.7, CI: 4.6–7.0, *p* = 0.000**	HR: 5.9, CI: 4.3–7.9, *p* = 0.000**	HR: 5.6, CI: 4.2–7.5, *p* = 0.000**		
BMI ≥ 30	HR: 1.0, CI: 0.8–1.3, *p* = 0.863	HR: 1.0, CI: 0.8–1.5, *p* = 0.675	HR: 1.0, CI: 0.7–1.3, *p* = 0.808	HR: 1.4, CI: 0.9–2.0, *p* = 0.117	HR: 0.9, CI: 0.7–1.2, *p* = 0.422
Diabetes type 2	HR: 1.1, CI: 0.8–1.5, *p* = 0.591	HR: 1.2, CI: 0.7–2.0, *p* = 0.554	HR: 1.1, CI: 0.7–1.7, *p* = 0.835	HR: 1.0, CI: 0.6–1.9, *p* = 0.887	HR: 1.1, CI: 0.7–1.6, *p* = 0.671
Gender	HR: 1.3, CI:1.1–1.6, *p* = 0.009**			HR: 1.3, CI: 0.9–1.9, *p* = 0.112	HR: 1.3, CI: 1.0–1.7, *p* = 0.025*
Hypertension	HR: 1.2, CI: 1.0–1.5, *p* = 0.033*	HR: 1.4, CI: 1.1–1.9, *p* = 0.012*	HR: 1.1, CI: 0.9–1.5, *p* = 0.371	HR: 1.2, CI: 0.8–1.7, *p* = 0.444	HR: 1.3, CI: 1.0–1.6, *p* = 0.057
History of AMI	HR: 1.1, CI: 0.8–1.5, *p* = 0.540	HR: 1.4, CI: 0.7–2.7, *p* = 0.287	HR: 1.0, CI: 0.7–1.4, *p* = 0.996	HR: 1.3, CI: 0.7–2.6, *p* = 0.397	HR: 1.0, CI: 0.7–1.5, *p* = 0.813
History of stroke	HR: 1.9, CI: 1.3–2.9, *p* = 0.002**	HR: 1.5, CI: 0.8–2.8, *p* = 0.225	HR: 2.3, CI: 1.3–3.9, *p* = 0.004**	HR: 1.8, CI: 0.9–3.5, *p* = 0.088	HR: 1.9, CI: 1.1–3.2, *p* = 0.013*
Periodontitis	HR: 1.4, CI: 1.2–1.8, *p* = 0.002**	HR: 1.4, CI: 1.0–1.9, *p* = 0.055	HR: 1.5, CI: 1.1–1.9, *p* = 0.006**	HR: 2.2, CI: 1.5–3.2, *p* = 0.000**	HR: 1.2, CI: 1.0–1.6, *p* = 0.102
Smoking	HR: 1.0, CI: 0.8–1.2, *p* = 0.977	HR: 0.9, CI: 0.6–1.2, *p* = 0.333	HR: 1.1, CI: 0.9–1.5, *p* = 0.321	HR: 1.1, CI: 0.8–1.5, *p* = 0.624	HR: 1.0, CI: 0.8–1.2, *p* = 0.810

*AMI* acute myocardial infarction, *BMI* body mass index, Periodontitis = alveolar bone loss ≥ 5-mm distance from the CEJ to marginal bone level on ≥ 30% of sites, *YO* young-old 60–72 years, *OO* old-old 78–93 years, *p < 0.05, **p < 0.01

A significant association was found between periodontitis and the number of lost teeth (OR: 1.1, CI: 1.1–1.1, *p* = 0.000) using binary logistic regression analysis. In a second Cox regression analysis, the number of lost teeth at baseline was used as a proxy for periodontitis with the number of lost teeth as an independent variable and incidence of the first event of a stroke or ischemic heart diseases as the dependent variable and with adjustment for the following variables: age group, BMI ≥ 30, diabetes type 2, gender, hypertension, history of acute myocardial infarction, history of stroke, and smoking. The number of lost teeth increased the risk for ischemic heart diseases in all individuals (HR: 1.0, CI: 1.0–1.1, *p* = 0.006), in women (HR: 1.0, CI: 1.0–1.1, *p* = 0.019), and in the YO group (HR: 1.0, CI: 1.0–1.1, *p* = 0.006) (Table 5). No significant association was identified between the number of lost teeth and stroke, in neither of all individuals, women, men, YO, and OO (Table 6). Using the number of lost teeth as an independent variable and incidence of mortality as the dependent variable adjusting for the same variables as above, the number of lost teeth increased the risk for mortality in all individuals (HR: 1.0, CI: 1.0–1.0, *p* = 0.000) and in women (HR: 1.0, CI: 1.0–1.0, *p* = 0.000) (Table 7).

**Table 5:** Associations between ischemic heart diseases and different independent variables including the number of lost teeth by Cox regression analysis

Independent variables at baseline	All individuals (*n* = 813) HR, CI, *p* value	Women (*n* = 430) HR, CI, *p* value	Men (*n* = 383) HR, CI, *p* value	YO (*n* = 452) HR, CI, *p* value	OO (*n* = 361) HR, CI, *p* value
Age cut	HR: 1.7, CI: 1.2–2.3, *p* = 0.002**	HR: 2.2, CI: 1.4–3.4, *p* = 0.001**	HR: 1.3, CI: 0.8–2.0, *p* = 0.291		
BMI ≥ 30	HR: 1.2, CI: 0.8–1.6, *p* = 0.356	HR: 1.1, CI: 0.7–1.8, *p* = 0.686	HR: 1.3, CI: 0.8–2.1, *p* = 0.320	HR: 1.2, CI: 0.8–1.9, *p* = 0.370	HR: 1.1, CI: 0.7–1.9, *p* = 0.666
Diabetes type 2	HR: 1.3, CI: 0.8–2.1, *p* = 0.257	HR: 1.4, CI: 0.7–2.8, *p* = 0.379	HR: 1.3, CI: 0.7–2.5, *p* = 0.427	HR: 1.3, CI: 0.7–2.5, *p* = 0.380	HR: 1.2, CI: 0.6–2.5, *p* = 0.571
Gender	HR: 1.2, CI: 0.9–1.7, *p* = 0.201			HR: 1.5, CI: 1.0–2.3, *p* = 0.036*	HR: 1.0, CI: 0.6–1.6, *p* = 0.995
Hypertension	HR: 1.5, CI: 1.1–2.0, *p* = 0.012*	HR: 1.8, CI: 1.2–2.8, *p* = 0.008**	HR: 1.2, CI: 0.7–1.8, *p* = 0.514	HR: 1.5, CI: 1.0–2.3, *p* = 0.072	HR: 1.5, CI: 0.9–2.3, *p* = 0.090
History of AMI	HR: 2.2, CI: 1.4–3.5, *p* = 0.000**	HR: 2.4, CI: 1.0–5.5, *p* = 0.040*	HR: 2.3, CI: 1.4–3.9, *p* = 0.002**	HR: 2.2, CI: 1.1–4.4, *p* = 0.03*	HR: 2.2, CI: 1.3–3.9, *p* = 0.007**
Smoking	HR: 1.1, CI: 0.8–1.5, *p* = 0.567	HR: 1.1, CI: 0.7–1.7, *p* = 0.700	HR: 1.2, CI: 0.8–1.8, *p* = 0.494	HR: 1.3, CI: 0.9–2.0, *p* = 0.202	HR: 0.9, CI: 0.6–1.5, *p* = 0.690
Tooth loss	HR: 1.0, CI: 1.0–1.1, *p* = 0.006**	HR: 1.0, CI: 1.0–1.1, *p* = 0.019**	HR: 1.0, CI: 1.0–1.1, *p* = 0.161	HR: 1.0, CI: 1.0–1.1, *p* = 0.006**	HR: 1.0, CI: 1.0–1.0, *p* = 0.285

*AMI* acute myocardial infarction, *BMI* body mass index, Tooth loss = number of lost teeth, *YO* young-old 60–72 years, *OO* old-old 78–93 years, *p < 0.05, **p < 0.01

**Table 6:** Associations between stroke and different independent variables including the number of lost teeth by Cox regression analysis

Independent variables at baseline	All individuals (*n* = 817) HR, CI, *p* value	Women (*n* = 431) HR, CI, p value	Men (*n* = 385) HR, CI, *p* value	YO (*n* = 447) HR, CI, *p* value	OO (*n* = 366) HR, CI, *p* value
Age cut	HR: 3.3, CI: 2.1–5.1, *p* = 0.000**	HR: 3.2, CI: 1.7–6.0, *p* = 0.000**	HR: 3.4, CI: 1.9–6.2, *p* = 0.000**		
BMI ≥ 30	HR: 0.7, CI: 0.4–1.1, *p* = 0.140	HR: 0.7, CI: 0.3–1.3, *p* = 0.256	HR: 0.8, CI: 0.4–1.5, *p* = 0.454	HR: 1.0, CI: 0.5–2.0, *p* = 0.961	HR: 0.5, CI: 0.3–1.1, *p* = 0.088
Diabetes type 2	HR: 0.8, CI: 0.4–1.7, *p* = 0.542	HR: 0.6, CI: 0.2–2.1, *p* = 0.422	HR: 1.0, CI: 0.4–2.6, *p* = 0.958	HR: 0.4, CI: 0.1–1.8, *p* = 0.232	HR: 1.1, CI: 0.5–2.7, *p* = 0.754
Gender	HR: 1.3, CI: 0.9–2.0, *p* = 0.151			HR: 1.2, CI: 0.7–2.2, *p* = 0.554	HR: 1.3, CI: 0.8–2.3, *p* = 0.272
Hypertension	HR: 1.7, CI: 1.2–2.6, *p* = 0.007**	HR: 2.0, CI: 1.1–3.5, *p* = 0.020*	HR: 1.6, CI: 0.9–2.7, *p* = 0.129	HR: 1.6, CI: 0.8–3.1, *p* = 0.147	HR: 1.8, CI: 1.1–3.1, *p* = 0.021*
History of stroke	HR: 3.6, CI: 1.9–6.6, *p* = 0.000**	HR: 2.5, CI: 0.9–6.9, *p* = 0.070	HR: 4.6, CI: 2.1–10.1, *p* = 0.000**	HR: 6.1, CI: 2.6–14.1, *p* = 0.000**	HR: 2.2, CI: 0.9–5.8, *p* = 0.103
Smoking	HR: 1.0, CI: 0.7–1.5, *p* = 0.909	HR: 0.8, CI: 0.4–1.4, *p* = 0.353	HR: 1.2, CI: 0.7–2.2, *p* = 0.483	HR: 1.2, CI: 0.7–2.2, *p* = 0.535	HR: 1.0, CI: 0.6–1.6, *p* = 0.881
Tooth loss	HR: 1.0, CI: 1.0–1.0, *p* = 0.593	HR: 1.0, CI: 1.0–1.1, *p* = 0.249	HR: 1.0, CI: 1.0–1.0, *p* = 0.717	HR: 1.0, CI: 1.0–1.1, *p* = 0.552	HR: 1.0, CI: 1.0–1.0, *p* = 0.921

*BMI* body mass index, Tooth loss = number of lost teeth, *YO* young-old 60–72 years, *OO* old-old 78–93 years, *p < 0.05, **p < 0.01

**Table 7:** Associations between mortality and different independent variables including the number of lost teeth by Cox regression analysis

Independent variables at baseline	All individuals (*n* = 810) HR, CI, *p* value	Women (*n* = 427) HR, CI, *p* value	Men (*n* = 383) HR, CI, *p* value	YO (*n* = 448) HR, CI, *p* value	OO (*n* = 362) HR, CI, *p* value
Age cut	HR: 5.0, CI: 4.0–6.2, *p* = 0.000**	HR: 4.8, CI: 3.5–6.6, *p* = 0.000**	HR: 5.2, CI: 3.8–7.1, *p* = 0.000**		
BMI ≥ 30	HR: 1.0, CI: 0.8–1.2, *p* = 0.913	HR: 1.0, CI: 0.7–1.4, *p* = 0.839	HR: 0.9, CI: 0.7–1.3, *p* = 0.658	HR: 1.3, CI: 0.9–1.9, *p* = 0.219	HR: 0.9, CI: 0.6–1.2, *p* = 0.347
Diabetes type 2	HR: 1.0, CI: 0.7–1.3, *p* = 0.806	HR: 1.0, CI: 0.6–1.8, *p* = 0.815	HR: 0.9, CI: 0.6–1.4, *p* = 0.641	HR: 0.8, CI: 0.4–1.5, *p* = 0.528	HR: 1.0, CI: 0.7–1.5, *p* = 0.943
Gender	HR: 1.4, CI: 1.1–1.7, *p* = 0.002**			HR: 1.5, CI: 1.0–2.1, *p* = 0.022	HR: 1.4, CI: 1.1–1.7, *p* = 0.014
Hypertension	HR: 1.2, CI: 1.0–1.5, *p* = 0.040*	HR: 1.4, CI: 1.0–1.9, *p* = 0.025*	HR: 1.1, CI: 0.8–1.5, *p* = 0.393	HR: 1.1, CI: 0.8–1.6, *p* = 0.597	HR: 1.3, CI: 1.0–1.6, *p* = 0.060
History of AMI	HR: 1.1, CI: 0.8–1.6, *p* = 0.403	HR: 1.5, CI: 0.8–2.9, *p* = 0.212	HR: 1.0, CI: 0.7–1.5, *p* = 0.813	HR: 1.2, CI: 0.6–2.3, *p* = 0.580	HR: 1.1, CI: 0.7–1.5, *p* = 0.710
History of stroke	HR: 1.7, CI: 1.1–2.5, *p* = 0.012*	HR: 1.3, CI: 0.7–2.4, *p* = 0.426	HR: 2.0, CI: 1.2–3.5, *p* = 0.013*	HR: 1.7, CI: 0.8–3.3, *p* = 0.149	HR: 1.8, CI: 1.1–2.9, *p* = 0.032
Smoking	HR: 1.0, CI: 0.8–1.2, *p* = 0.956	HR: 0.9, CI: 0.6–1.2, *p* = 0.337	HR: 1.2, CI: 0.9–1.5, *p* = 0.311	HR: 1.1, CI: 0.8–1.6, *p* = 0.555	HR: 1.0, CI: 0.8–1.2, *p* = 0.828
Tooth loss	HR: 1.0, CI: 1.0–1.0, *p* = 0.000**	HR: 1.0, CI: 1.0–1.1, *p* = 0.000**	HR: 1.0, CI: 1.0–1.0, *p* = 0.050	HR: 1.1, CI: 1.0–1.1, *p* = 0.000**	HR: 1.0, CI: 1.0–1.0, *p* = 0.090

*AMI* acute myocardial infarction, *BMI* body mass index, Tooth loss = number of lost teeth, *YO* young-old 60–72 years, *OO* old-old 78–93 years, *p < 0.05, **p < 0.01

## Discussion

The present study identified that over the 17-year follow-up period, periodontitis increased the risk of future incidences of ischemic heart diseases in all individuals, in women, and in the OO age group. In woman with periodontitis, the HR for the incidence of ischemic heart disease was 2.1. Another study has confirmed that after the menopause, women have a higher incidence of AMI compared to age-matched men [22] which is in agreement with the results of the present study in older people. There are few other longitudinal studies concerning periodontitis and incidences of CVDs. In a Danish national register–based cohort study with a follow-up period of 15 years, patients with periodontitis were reported to have an increased risk of CVDs [23]. In the study by Hansen et al. (2013), ICD codes were used to define periodontitis. This classification of periodontitis is different from the one used in the present study. Additionally, the age of the study population was > 18 years, and gender differences were not reported making comparisons between results observed in the present study impossible. A Korean nationwide cohort follow-up study of 7.6 years showed a dose-dependent association with tooth loss and incident myocardial infarction, heart failure, and ischemic stroke, especially in individuals with periodontitis [24]. The circumstances between the study above and our study are not the same. The classification of periodontitis was in the study by Lee et al. [24] not defined, and the study individuals included were from 20 years, and the mean age not mentioned. Also, the included individuals had a history of a CVD event, meaning that they were at an increased risk for a subsequent CVD [25].

Another main finding in this study was that individuals with periodontitis were at a higher risk to die during the 17-year follow-up compared to individuals without a diagnosis of periodontitis. Recently published data have shown that periodontitis increased the risk for all-cause mortality within 15 years [23]. In a 3-year follow-up study, individuals with severe periodontitis developed the combined endpoint (myocardial infarction, stroke/transient ischemic attack, cardiovascular death, and death caused by stroke) more often compared to individuals without periodontitis (18.9% versus 14.2%) [26]. As we were unaware of the exact reasons for death in the present study, such a combined endpoint was not possible to include. In the present study, it was demonstrated that in men and the YO (60–72 years), there was an association between periodontitis and mortality. Young individuals (30–40 years) with periodontitis and missing molars have been reported to have an increased risk for early death over 16 years [27]. Missing teeth, due to periodontal disease, could be a proxy for a previous inflammation and partly explain the identified association with an increased risk for mortality among young-old in the present study that is in line with the results from the study by Lee et al. [24]. In another recent study, ≥ nine missing teeth were also associated with mortality [28]. In the present study, an association between periodontitis and the number of lost teeth at baseline was found. When lost teeth at baseline were used as a proxy for periodontitis, an association between all-cause mortality in all individuals was found. Such an association is in line with the results obtained using periodontitis as the independent variable in the Cox regression analysis. However, lost teeth at baseline were also associated with mortality among women and to an increased risk for ischemic heart diseases in all individuals, in women, and in the YO group. Accordingly, using lost teeth as a proxy for periodontitis does not seem to be an adequate approximation for periodontal disease.

Deaths by CVDs have decreased during the latest 60 years, as a result of preventive care and advances in medicine [29]. Many older individuals are using preventive medications that may delay or even prevent a CVD event from occurring [30].

Recent data reported that the numbers of missing teeth were related to heart failure and myocardial infarction. In contrast, the number of teeth missing was not significantly related to stroke in a longitudinal study with a median follow-up time of 15.8 years [31]. In another study, the same tendency was reported; missing teeth (≥ five missing teeth) were statistically associated with an event of coronary heart disease and acute myocardial infarction. In contrast, tooth loss was not associated with stroke in a 13-year prospective longitudinal study [28].

In the literature, different classifications and parameters for periodontitis have been used. Missing teeth have also been proposed as a proxy for current or past periodontitis as it is considered to reflect an accumulation of oral inflammation [32]. Correctly, if untreated, periodontitis may result in tooth loss and is one of the primary reasons for tooth loss in adulthood [33]. In a recent study by Lee et al. [24], edentulous individuals demonstrated the highest cardiovascular risk. It is, however, difficult to be sure of the reason for missing teeth unless it is reported in the dental records. In the present study, it was not possible to within certainty decide the reason for tooth loss. Alveolar bone loss, as used in the present study as a proxy for periodontal disease, is an indicator of a patient who has had periodontal inflammation during a period.

The prevalence of periodontitis defined by loss of alveolar bone was relatively low (24.7%). The reported prevalence of periodontitis in individuals 65 years and older has been reported to be 70% in the USA and with increasing prevalence with increasing age [3]. Differences in prevalence may be related to the different classifications of periodontitis used. Eke et al. [3], when defining “total periodontitis,” included mild, moderate, and severe stages of periodontitis and used clinical attachment–level loss as an indicator for periodontitis. The definition of periodontitis used in the present study is based on bone loss of ≥ 5 mm from the CEJ to the alveolar bone level on ≥ 30% of sites. The use of the 5-mm level was chosen to account for possible measurement errors. Individuals defined as mild periodontitis cases in the study by Eke et al. [3] were not defined as periodontitis patients in the present study, which to some degree may explain the differences in prevalence figures reported in the two studies. Individuals affected with bone loss in older ages may reflect a long history of periodontitis and accordingly a long time of an inflammatory response.

The new accepted classification of periodontitis includes clinical variables (CAL and probing pocket depth) as well as bone loss in radiographs [34]. The bone loss reflects the accumulated progressive effect of periodontitis over a long time [35, 36]. Clinical parameters partly reflecting the inflammatory activity could be more transient, giving the information of the periodontal status at that specific moment. Studies have reported that the mean proportion of bone loss increased with age, but the proportion of teeth with periodontal pockets remained unmodified. In another study, the alveolar bone loss progressed with age but was limited after the age of 50 [37]. In older individuals, gingival recession is the main reason for attachment loss [38]. Crestal bone height and CAL have shown a good correlation [39]. It has been proven that attachment loss precedes radiographic evidence of crestal alveolar bone loss during periods of periodontal disease activity [40] whereas, over time, these differences seem to level off [39]. In our study, the limit of alveolar bone loss ≥ 5-mm distance from CEJ to marginal bone level reflects a definitive bone loss and such a bone loss is present on ≥ 30% of sites corresponding to a general disease distribution.

One limitation with the present study was that the most fragile and medically compromised individuals were not able to participate. The fact that the most fragile individuals did not participate may have affected the results, possibly lowering the associations between periodontitis and CVDs. The individuals in the present study were 60 years or older. While the follow-up period was long, obviously many died during the study. The causes of death were not known, but the main reasons for death are still CVDs [41]. The consequence of not knowing the reasons for death makes it impossible to include mortality by CVDs in a combined endpoint, among others, which should have to strengthen the associations between periodontitis and CVDs. The risk factors in the Cox regression analysis were adjusted only from the baseline data, which is another limitation in the study.

The long-time follow-up and that CVD events were easy to follow and control is a strength with the present study. It would be interesting to study if well-designed preventive dental programs can influence the incidence of CVDs in long-term studies. Intervention studies are needed to verify a valid link between periodontitis and CVDs.

In conclusion, this study demonstrated that in older adults, periodontitis was a statistical risk indicator for ischemic heart diseases. Over the time studied, periodontitis was significantly associated with mortality. This is the first long-time follow-up report on periodontitis and the incidence of cardiovascular diseases and death.
